# Socioeconomic inequalities and COVID-19 – A review of the current international literature. Additional material: Overview of the included publications

**DOI:** 10.25646/7134

**Published:** 2020-10-09

**Authors:** Benjamin Wachtler, Niels Michalski, Enno Nowossadeck, Michaela Diercke, Morten Wahrendorf, Claudia Santos-Hövener, Thomas Lampert, Jens Hoebel

**Affiliations:** 1 Robert Koch Institute, Berlin Department of Epidemiology and Health Monitoring; 2 Robert Koch Institute, Berlin Department of Infectious Disease Epidemiology; 3 University of Düsseldorf, Medical Faculty, Institute of Medical Sociology, Centre for Health and Society

**Table:** Socioeconomic inequalities and COVID-19 – A review of the current international literature. Additional material: Overview of the included publications* Source: Own table

**Peer-reviewed articles**	**#**	**Author**	**Title**	**Country**	**Date published**	**Study design**	**Study population**	**Study period**	**Measures of Socioeconomic Status**	**Outcome measures**	**Results**
	1	Azar et al. [41]	Disparities In Outcomes Among COVID-19 Patients In A Large Health Care System In California	USA	21.05.2020	Retrospective cohort	14,036 tested, 1,052 confirmed cases of one integrated health care system in Northern California	01.01. - 08.04.2020	Ethniciy/race, income, insurance plan	Incidence, hospitalisation	Individuals with Medicaid or who were self-pay or had no reported insurance had twice the odds of being admitted, compared to those with commercial insurance (OR=2.1 for both; p<0,05). COVID-19 positive patients residing in ZIP codes within the top two quartiles of income (quartiles 3 and 4) were less likely to be admitted to the hospital than those residing in the bottom-quartile ZIP code (OR=0.24 and 0.55 for the top two quartiles).
	2	Kim & Bostwick [65]	Social Vulnerability and Racial Inequality in COVID-19 Deaths in Chicago	USA	21.05.2020	Ecological study	269 deaths in Chicago		Ethnicity/race, Social Vulnerability Index (SVI)	Mortality	Community areas with higher levels of SVI and risk factor score had a significantly higher COVID death rate. For the most part, the effect of social vulnerability on the death rate was medicated through health risk factors.
	3	Lassale et al. [68]	Ethnic disparities in hospitalisation for COVID-19 in England: The role of socioeconomic factors, mental health, and inflammatory and proinflammatory factors in a community-based cohort study	England	01.06.2020	Retrospective cohort	340,966 participants, 640 cases	16.03. - 26.04.2020	Self-reported ethnicity, Townsend index of area deprivation, household income, educational attainment, occupation	Hospitalisation	After adjusting for age and sex, compared to White participants, being from a Black ethnic background was associated with over a four-fold risk of hospitalisation for COVID-19 (odds ratio; 95% confidence interval: 4.32; 3.00–6.23), while a doubling was apparent in Asian (2.12; 1.37, 3.28) and other ethnic groups (1.84; 1.13, 2.99). The greatest attenuations were observed when socioeconomic factors were added to the multivariable model: 24.5% for Blacks, 31.9.3% for Asians, and 30.0% for others.
	4	Millett et al. [18]	Assessing Differential Impacts of COVID-19 on Black Communities	USA	14.05.2020	Ecological study	US population	Until 13.04.2020	Ethnicity/race, insurance, household crowding, unemployment	Incidence, mortality	Higher rates of COVID-19 cases were independently associated with greater proportions of uninsured residents (RR 1.16, 95% CI 1.07-1.126), higher percentages of residents in crowded living conditions (RR 1.05, 95% CI 1.01-1.10), and more days since the first case (RR 3.1, 95% CI 2.9-3.3). 280,112 cases of excess COVID-19 diagnoses were associated with occupancy of greater than one person per room, and 126,985 excess diagnoses of COVID-19 were associated with lack of health insurance. The population attributable fraction (PAF) for lack of health insurance was 3.3% for counties with <13% black residents and 4.2% for counties with >13% black residents. Higher county-level unemployment was associated with fewer COVID-19 diagnoses.
	5	Mollalo et al. [42]	GIS-based spatial modeling of COVID-19 incidence rate in the continental United States	USA	22.04.2020	Ecological study	US population	22.01. - 09.04.2020	Median household income, income inequalities, percentage uninsured, unemployment rate, food insecurity	Incidence	Income inequality, median household income, the percentage of nurse practitioners, and the percentage of the black female population (to the total female population) at the county-level were positively associated with the COVID-19 incidence rate on county level.
	6	Niedzwiedz et al. [67]	Ethnic and socioeconomic differences in SARS-CoV-2 infection: prospective cohort study using UK Biobank	England	29.05.2020	Retrospective cohort	392,116 participants in England, 2,658 tested, 948 cases, 726 in hospital	16.03-03.05.2020	Ethnicity/race, occupation, Townsend Index of area deprivation, educational level, employment status		Living in a disadvantaged area (according to the Townsend deprivation score) was associated with a higher risk of confirmed infection, particularly for the most disadvantaged quartile (RR 2.19 (95% CI 1.80–2.66)). Differences in ethnicity and country of birth, social factors, baseline health and behavioural risk factors all moderately attenuated the association in the most disadvantaged quartile.
	7	Okoh et al. [83]	Coronavirus disease 19 in minority populations of Newark, New Jersey	USA	10.06.2020	Retrospective cohort	African Americans and Hispanics admitted to one hospital in Newark, n = 416 cases	10.03. - 10.04.2020	Population densities, housing units and median income on zip code level	Hospitalisation, mortality	Minority patients admitted for COVID-19 were in the highest quartile for population’s density, number of housing units and disproportionately fell into the lowest median income quartile.
	8	Price-Haywood et al. [40]	Hospitalization and Mortality among Black Patients and White Patients with Covid-19	USA	27.05.2020	Retrospective cohort	3,481 cases	01.03. - 11.04.2020	Ethnicity/race, insurance plan, zip code level income	Length of hospital stay, mortality	Increasing age, a higher score on the Charlson Comorbidity Index, public insurance (Medicare or Medicaid), residence in a low-income area, and obesity were associated with an increased odds of admission. However, black race was not independently associated with risk of in-hospital death (hazard ratio, 0.89; 95% CI, 0.68 to 1.17).
	9	Ramirez & Lee [53]	COVID-19 Emergence and Social and Health Determinants in Colorado: A Rapid Spatial Analysis	USA	29.05.2020	Ecological study	Population of Colorado	14.03. - 08.04.2020	Social vulnerability index (SVI)	Mortality	Higher COVID-19-related death rates were population density and asthma hospitalization, suggestive of urban areas, and poverty and unemployment, suggestive of rural areas. Furthermore, a spatial overlap of high rates of chronic diseases with high rates of COVID-19 may suggest a broader syndemic health burden, where comorbidities intersect with inequality of social determinants of health.
	10	Wadhera et al. [54]	Variation in COVID-19 Hospitalizations and Deaths Across NewYork City Boroughs	USA	29.04.2020	Ecological study	Population of New York City	Until 25.04.2020	Median income, Education	Incidence, hospitalisation, mortality	The number of patients with COVID-19 who were hospitalized per 100,000 population was highest in the Bronx (634) and lowest in Manhattan (331). The number of deaths related to COVID-19 per 100,000 population was also highest in the Bronx (224) and lowest in Manhattan (122). Household median income was lowest in the Bronx ($38,467) as was the proportion of persons with a bachelor’s degree or higher (20.7%). There were 48 short-term acute care hospitals. The number of hospitals per borough ranged from 2 in Staten Island to 16 in Manhattan. The number of hospital beds per 100,000 population was lowest in Queens (144 beds) and highest in the Bronx (336 beds) and in Manhattan (534 beds).
Not peer-reviewed publications	11	Abedi et al. [43]	Racial, Economic and Health Inequality and COVID-19 Infection in the United States	USA	01.05.2020	Ecological study	Population of 369 counties (total population: 102,178,117 [median: 73,447])	05.04. - 16.04.2020	Ethnicity/race, poverty level, median income, education, rate of uninsured population	Incidence, mortality	Counties with more diverse demographics, higher population, education, income levels, and lower disability rates were at a higher risk of COVID-19 infection. Counties with higher disability and poverty rates had a higher death rate. African Americans were more vulnerable to COVID-19 than other ethnic groups (1,981 African American infected cases versus 658 Whites per million).
	12	Ahmad et al. [81]	Association of Poor Housing Conditions with COVID-19 Incidence and Mortality Across US Counties.	USA	30.05.2020	Ecological study	3,141 US counties		Poor housing conditions, median household income, education, ethnicity	Incidence	In the fully adjusted models, with each 5% increase in percent households with poor housing conditions, there was a 50% higher risk of COVID-19 incidence (IRR 1.50, 95% CI: 1.38 – 1.62) and a 42% higher risk of COVID-19 mortality (MRR 1.42, 95% CI: 1.25 – 1.61). Results remained similar using earlier timepoints (3/31/2020 and 4/10/2020).
	13	Apea et al. [69]	Ethnicity and outcomes in patients hospitalised with COVID-19 infection in East London: an observational cohort study	England	12.06.2020	Retrospective cohort	1,737 patients admitted to five acute NHS Hospitals in East London	01.03. - 13.05.2020	Ethnicity, Index of Multiple Deprivation	Mortality, admission to intensive care	The majority of patients were classified as being in the two most deprived socio-economic quintiles in England.
	14	Buja et al. [62]	Demographic and Socio-Economic Factors, and Healthcare Resource Indicators Associated with the Rapid Spread of COVID-19 in Northern Italy: An Ecological Study	Italy	29.04.2020	Ecological study	Population of 36 provinces in Northern Italy	24.02. - 30.03.2020	Regional employment rates	Incidence	A significant positive correlation was found with employment rates, public transportation rates, in-house density, population density, and the proportions of private acute and long-term care beds in clinics and nursing homes.
	15	Chow et al. [44]	The disproportionate rise in COVID-19 cases among Hispanic/Latinx in disadvantaged communities of Orange County, California: A socioeconomic case-series	USA	07.05.2020	Ecological study	154 cases	12.03. - 22.04.2020	Ethnicity/race, regional household income, education, health insurance status	Incidence	Hispanic/Latinx patients residing in census tracts below the median income demonstrated exponential growth (rate = 55.9%, R2 = 0.9742) during the study period. In addition, there was a significant difference for both race-ethnic (p < 0.001) and income bracket (p = 0.001) distributions prior to and after California’s shelter-in-place. In addition, the percentage of individuals residing in neighborhoods with denser households (p = 0.046), lower levels of college graduation (p < 0.001), health insurance coverage (p = 0.01), and ability to work from home (p < 0.001) significantly increased over the same timeframe.
	16	Cyrus et al. [55]	The impact of COVID-19 on African American communities in the United States	USA	19.05.2020	Ecological study	152 counties	22.01. - 12.04.2020	Poverty level	Incidence, mortality	COVID-19 prevalence increased 5% for every percentage increase in county African American density, and the death rate increased 2 per hundred thousand for every percentage increase in county African American density.
	17	Federgruen & Naha [56]	Variation in Covid-19 Cases Across New York City	USA	29.05.2020	Ecological study	Population of New York City		Poverty Level	Incidence	An increase of the average household size by one member accounts, in our final model specification, for at least 876 cases, a full 23% of the span of incidence numbers, at a 95% confidence level. The percentage of the population above the age of 65, as well as that below the poverty line, are additional indicators with a significant impact on the case incidence rate, along with their interaction term.
	18	FieldingMiller et al. [57]	Social determinants of COVID-19 mortality at the county level	USA	08.05.2020	Ecological study	2,743 counties across 50 states		Poverty level, rate uninsured	Mortality	In urban counties (n=114), only population density was significantly associated with COVID-19 mortality (b = 0.21, p <0.001). In non-urban counties (n=2,629), all hypothesized social determinants were significantly associated with higher levels of mortality. Percentage of uninsured individuals was associated with lower reported COVID-19 mortality (b = -0.36, p = 0.001).
	19	Guha et al. [45]	Community and Socioeconomic Factors Associated with COVID-19 in the United States: Zip code level cross sectional analysis	USA	22.04.2020	Ecological study	Population of New York City, Chicago, Richmond county of Detroit, Kings County of Seattle, Miami-Dade County	Until 11.04.2020	Ethnicity/race, household income, poverty level, median house value, education	Incidence, mortality	A multivariable linear regression model noted that 1% increase in the proportion of residents above the age of 65 years, proportion of African American residents, proportion of females, persons per household and population density of the zip code increased the proportion of positive cases by 0.77%, 0.23%, 1.64%, 1.83% and 0.46% respectively (P<0.01) with only population density remaining significant in zip codes with greater than median number of cases. In zips with greater than median number of deaths, no community/socio-economic factor contributed significantly to death.
	20	Ho et al. [70]	Modifiable and non-modifiable risk factors for COVID-19: results from UK Biobank	England	02.05.2020	Retrospective cohort	428,225 participants, 340 cases	16.03. - 14.04.2020	Ethnicity/race, Townsend index of area deprivation	Incidence	Non-modifiable risk factors included older age (RR 1.10 per 5 years), male sex (RR 1.64), black ethnicity (RR 1.86)and socioeconomic deprivation (RR 1.26 per SD increase in Townsend Index).
	21	Khanijahania [82]	County-Level Proportions of Black and Hispanic populations, and Socioeconomic Characteristics in Association with Confirmed COVID-19 Cases and Deaths in the United States	USA	05.06.2020	Ecological study	3,142 US counties in 50 states		Ethnicity/race, household income, education, financial hardship, insurance coverage, unemployment	Incidence, mortality	US counties with a higher proportion of black population, and a higher proportion of adults with less than high school diploma had disproportionately higher COVID-19 cases and deaths (ß>0, p<0.05). A higher proportion of the Hispanic population was associated with higher confirmed cases (ß= 1.03, 95% CI= 0.57-1.5), and higher housing cost to household income ratio was associated with higher deaths (ß= 3.74, 95% CI= 2.14-5.37).
	22	Khawaja et al. [71]	Associations with covid-19 hospitalisation amongst 406,793 adults: the UK Biobank prospective cohort study	England	11.05.2020	Prospective cohort study	406,793 participants, 605 cases	16.03. - 16.04.2020	Ethnicity/race, residential deprivation	Hospitalisation	Major independent risk factors for hospitalisation with covid-19 were male sex (odds ratio 1.52; 95% confidence interval 1.28 to 1.81; P<0.001), South Asian ethnicity (2.02; 1.28 to 3.17; P=0.002) or black ethnicity (3.09; 2.18 to 4.38; P<0.001) compared to white ethnicity and greater residential deprivation (1.92 for most deprived quartile compared to least deprived quartile; 1.50 to 2.47; P<0.001).
	23	Li et al. [46]	Multivariate Analysis of Factors Affecting COVID-19 Case and Death Rate in U.S. Counties: The Significant Effects of Black Race and Temperature	USA	24.04.2020	Ecological study	661 counties	Until 14.04.2020	Ethnicity, poverty level, violence rate	Incidence, mortality	Risk factors associated with increased cases and/or deaths per 100,000 included increased GDP per capita, increased percent Black, decreased percent Hispanic, decreased percent Asian, increased poverty and decreased access to primary care. Multivariate regression analyses demonstrated Black race is a risk factor for worse COVID-19 outcome independent of comorbidities, poverty, access to health care, and other mitigating factors.
	24	Liu et al. [72]	Time courses of COVID-19 infection and local variation in socioeconomic and health disparities in England	England	29.05.2020	Ecological study	Population of England	30.01. - 06.05.2020	Ethnicity, Index of Multiple Deprivation	Incidence	The higher case trajectory cluster (79 local authorities) had higher proportion of Black and Asian residents (p=0.03; 29 p=0.02), higher multiple deprivation scores (p<0.001). Local authorities with higher proportions of Black residents were more likely to belong to the high case trajectory cluster, even after adjusting for population density, deprivation, proportion of older adults and preventable mortality (p=0.04).
	25	Maroko et al. [63]	Covid-19 and Inequity: A comparative spatial analysis of New York City and Chicago hot spots	USA	24.04.2020	Ecological study	Population of New York City and Chicago	Until 13.04.2020	Household density, ethnicity/race, education, household income, poverty level, unemployment rate, occupation	Incidence	In both cities, cold spots (clusters of low SARS-CoV-2 rate) demonstrated typical protective factors associated with the social determinants of health and the ability to social distance. These neighborhoods tended to be wealthier, have higher educational attainment, higher proportions of non-Hispanic white residents, and more workers in managerial occupations. Hot spots (clusters of high SARS-CoV-2 rate) also had similarities, such as lower rates of college graduates and higher proportions of people of color. It also appears to be larger households (more people per household), rather than overall population density, that may to be a more strongly associated with hot spots.
	26	Mukherji [47]	The Social and Economic Factors Underlying the Impact of COVID-19 Cases and Deaths in US Counties	USA	19.05.2020	Ecological study	Population of 771 counties	30.03. - 18.04.2020	Regional income, income inequality, poverty level, unemployment rate	Incidence, mortality	The paper finds that counties with high per capita personal income have high incidence of both reported cases and deaths. The unemployment rate is negative for deaths implying that places with low unemployment rates or higher economic activity have higher reported deaths. Counties with higher income inequality as measured by the Gini coefficient experienced more deaths and reported more cases. Counties with high concentrations of non-Hispanic Blacks, Native Americans, and immigrant populations have higher incidence of both cases and deaths.
	27	Nayak et al. [73]	Impact of Social Vulnerability on COVID-19 Incidence and Outcomes in the United States	USA	17.04.2020	Ecological study	Population of 433 counties, 283,256 cases and 6,644 deaths	Until 04.04.2020	Social Vulnerability Index (SVI)	Incidence, case-fatality-rate	Higher SVI, indicative of greater social vulnerability, was associated with higher CFR (RR: 1.19 [1.05, 1.34], p=0.005, per-1 unit increase), an association that strengthened after adjustment for age>65 years and comorbidities (RR: 1.63 [1.38, 1.91], p<0.001), and was further confirmed in a sensitivity analysis limited to six states with the highest testing levels. Although the association between overall SVI and COVID-19 incidence was not significant, the SVI sub-components of socioeconomic status and minority status were both predictors of higher incidence and CFR. A combination of high SVI (>0.46) and high adjusted CFR (>2.3%) was observed in 28.9% of counties.
	28	Nazroo et al. [74]	Evidence for ethnic inequalities in mortality related to COVID-19 infections: Findings from an ecological analysis of England and Wales	England and Wales	09.06.2020	Ecological study	Population of England and Wales	Until 24.04.2020	Index of Multiple Deprivation, ethnicity	Mortality	For every 1% rise in proportion of the population who are ethnic minority, COVID-19 related deaths increased by 5·10 (3·99 to 6·21) per million. This rise is present for each ethnic minority category examined. The size of this increase is a little reduced in a fully adjusted model, suggesting that some of the association results from ethnic minority people living in more densely populated, more polluted and more deprived areas.
	29	Patel et al. [75]	Race, Socioeconomic Deprivation, and Hospitalization for COVID-19 in English participants of a National Biobank	England	02.05.2020	Prospective cohort study	418,794 participants		Ethnicity, Townsend Index of Area Deprivation	Hospitalisation	COVID-19 hospitalization was noted in 32 of 7,714 (0.4%) black participants, 28 of 10,614 (0.2%) Asian participants, and 489 of 400,438 (0.1%) white participants, with results largely consistent across English regions In a logistic regression model adjusted for age, sex, and geographic region, both black participants (odds ratio 3.4; 95%CI 2.4-4.9) and Asian participants (odds ratio 2.1; 95%CI 1.5-3.2) were at increased risk as compared to white participants. The relationship between race and COVID-19 hospitalization was only modestly attenuated in a logistic regression model that additionally adjusted for Townsend Deprivation Index and household income--odds ratios for black and Asian participants of 3.1 (95%CI 2.0-4.8) and 2.0 (95%CI 1.2-3.1) as compared to white participants respectively.
	30	Plümper & Neumayer [48]	The COVID-19 Pandemic Predominantly Hits Poor Neighborhoods, or does it? Evidence from Germany	Germany	18.05.2020	Ecological study	German population	Until 13.04. and 14.04. - 17.05.2020	Regional income, education, rate of social welfare benefits	Incidence	First time period: Higher incidence in regions with high income, high education, low rate of social welfare, in second time period high income and education are associated with lower incidence rates.
	31	Prats-Uribe et al. [76]	Ethnicity, comorbidity, socioeconomic status, and their associations with COVID-19 infection in England: a cohort analysis of UK Biobank data	England	08.05.2020	Retrospective cohort	415,582 participants, 1,416 tested, 651 cases	Until 14.04.2020	Ethnicity, Index of Multiple Deprivation	Incidence	The incidence of COVID-19 was 0.61% (95% CI: 0.46%-0.82%) in Black/Black British participants, 0.32% (0.19%-0.56%) in ‘other’ ethnicities, 0.32% (0.23%-0.47%) in Asian/Asian British, 0.30% (0.11%-0.80%) in Chinese, 0.16% (0.06%-0.41%) in mixed, and 0.14% (0.13%-0.15%) in White. Compared with White participants, Black/Black British participants had an adjusted relative risk (RR) of 3.30 (2.39-4.55), Chinese participants 3.00 (1.11-8.06), Asian/Asian British participants 2.04 (1.36-3.07), ‘other’ ethnicities 1.93 (1.08-3.45), and mixed ethnicities 1.07 (0.40-2.86). Socioeconomic status (adjusted RR 1.93 (1.51-2.46) for the most deprived), obesity (adjusted RR 1.04 (1.02-1.05) per kg/m2) and comorbid hypertension, chronic obstructive pulmonary disease, asthma, and specific renal diseases were also associated with increased risk of COVID-19.
	32	Raisi-Estabragh et al. [19]	Greater risk of severe COVID-19 in non-White ethnicities is not explained by cardiometabolic, socioeconomic, or behavioural factors, or by 25(OH)-vitamin D status: study of 1,326 cases from the UK Biobank	United Kingdom	02.06.2020	Retrospective cohort study	497,996 participants, 4,510 tested, 1,326 cases	16.03. - 18.05.2020	Ethnicity/race, Index of Multiple Deprivation, housing	Incidence	There was over-representation of men and non-White ethnicities in the COVID-19 positive group. Non-White ethnicity, Townsend deprivation score and household overcrowding were independently associated with significantly greater odds of COVID-19. The pattern of association was consistent for men and women; socio-demographic and behavioural factors did not attenuate sex/ethnicity associations.
	33	Rose et al. [58]	Inequalities in COVID19 mortality related to ethnicity and socioeconomic deprivation	England	30.04.2020	Ecological study	Population of England	Until 22.04.2020	Ethnicity/race, poverty level	Mortality	Local authorities with a greater proportion of residents from ethnic minority backgrounds had statistically significantly higher COVID-19 mortality rates, as did local authorities with a greater proportion of residents experiencing deprivation relating to low income. After adjusting for income deprivation and other covariates, each percentage point increase in the proportion of the population from BAME backgrounds was associated with a 1% increase in the COVID19 mortality rate [IRR=1.01, 95%CI 1.01–1.02]. Each percentage point increase in the proportion of the population experiencing income deprivation was associated with a 2% increase in the COVID19 mortality rate [IRR=1.02, 95%CI 1.01–1.04].
	34	Sy et al. [49]	Socioeconomic disparities in subway use and COVID-19 outcomes in New York City	USA	30.05.2020	Ecological study	Population of New York City	Until 26.04.2020	Regional income, education, ethnicity, rate uninsured	Incidence	Increased subway use was associated with a higher rate of COVID-19 cases per 100,000 population when adjusted for testing effort (aRR=1.11; 95% CI: 1.03-1.19), but this association was weaker once we adjusted for median income (aRR=1.06; 95% CI: 1.00-1.12). All sociodemographic variables were significantly associated with the rate of positive cases per 100,000 population when adjusting for testing effort (except percent uninsured) and adjusting for both income and testing effort. The risk factor with the strongest association with COVID-19 was the percent of individuals in essential work (aRR = 1.59, 95% CI: 1.36-1.86).
	35	Takagi et al. [50]	Ethnics and economics in COVID-19: Meta-regression of data from countries in the New York metropolitan area	USA	24.05.2020	Ecological study	Population of 31 countries in the New York City Metropolitan Area	Until 20.05.2020	Ethnicity/race, poverty level, household income	Incidence, mortality	A slope (coefficient) of the univariable meta-regression line for COVID-19 prevalence was not significant for household income (P = .639), whereas the coefficient was significantly positive for black (coefficient, 0.021; P = .015), Hispanic/Latino (0.033; P < .001), and poverty (0.039; P = .02), which indicated that COVID-19 prevalence increased significantly as black, Hispanic/Latino, and poverty increased. The multivariable model revealed that the slope was significantly positive for only Hispanic/Latino (P < .001). The coefficient in the univariable model for COVID-19 fatality, however, was not significant for all the covariate.
	36	Takagi et al. [59]	Meta-regression of COVID-19 prevalence/fatality on socioeconomic characteristics of data from top 50 U.S. large cities	USA	27.05.2020	Ecological study	Population of 50 US cities	Until 22.05.2020	Ethnicity/race, education, household income, insurance rate, poverty level, unemployment rate	Incidence, mortality	COVID-19 prevalence was significantly negative for male sex, education attainment, computer and Internet use, and private health insurance. Whereas, the coefficient was significantly positive for black race, never matrimony, unemployment, and poverty. In the multivariable model, the coefficient was significantly negative for male sex (P = 0.036) and computer use (P = 0.024), and significantly positive for never matrimony (P < 0.001). A coefficient for COVID-19 fatality was significantly negative for no health insurance, and significantly positive for elderly, unemployment and public coverage.
	37	Vahidy et al. [51]	Racial and Ethnic Disparities in SARS-CoV-2 Pandemic: Analysis of a COVID-19 Observational Registry for a Diverse U.S. Metropolitan Population	USA	28.04.2020	Retrospective cohort	4,513 participants, 754 cases	05.03. - 12.04.2020	Ethnicity/race, household income	Incidence	Among 4,513 tested individuals, 754 (16.7%) tested positive. Overall mean (SD) age was 50.6 (18.9) years, 62% females and 26% were African American. African American race was associated with higher comorbidity burden, lower socio-economic status, and higher population density residence. In the fully adjusted model, African American race (vs. White; aOR, CI: 1.84, 1.49–2.27) and Hispanic ethnicity (vs. non-Hispanic; aOR, CI: 1.70, 1.35–2.14) had a higher likelihood of infection.
	38	Whittle & Diaz-Artiles [52]	An ecological study of socioeconomic predictors in detection of COVID-19 cases across neighborhoods in New York City	USA	22.04.2020	Ecological study	Population of New York City, 64,955 cases	Until 05.04.2020	Regional household income, unemployment rate, poverty level	Incidence	A decrease of $10,000 median household income is associated with a 2.5% (95% CI: 1.0% to 4.1% p = 0.002) increase in detected COVID-19 cases.
	39	Williamson et al. [77]	OpenSAFELY: factors associated with COVID-19-related hospital death in the linked electronic health records of 17 million adult NHS patients	England	07.05.2020	Retrospective cohort	17,425,445 people, 5,683 deaths	01.02. - 25.04.2020	Index of Multiple Deprivation, ethnicity	Mortality	Death from COVID-19 was strongly associated with being male (HR 1.99 (1.88-2.10)) older age and deprivation and comoibidities. Black people were at higher risk of death with only partial attenuatino in hazard ratios from the fully adjusted model (age-sex adjusted HR 2.17 (1.84-2.57), fully adjusted HR 1.71 (1.44-2.02) with similar findings for Asian people (age-adjusted HR 1.95 (1.73-2.18), fully adjusted HR 1.62 (1.43-1.82).
	40	Xie & Li [64]	Health and Demographic Impact on COVID-19 Infection and Mortality in US Counties	USA	11.05.2020	Ecological study	US population	21.01. - 23.04.2020	Regional poverty level, education, ethnicity/race, unemployment, household income, housing cost burden	Incidence	The higher percentage of adults with a high school or less education, the higher of COVID-19 infection rate in log scale (b = 0.02113, 95% CI: 0.01491, 0.02734). With the higher housing cost burden, the COVID-19 infection rate in log scale significantly increased (b = 0.03451, 95% CI: 0.01786, 0.05116). With the higher percentage of Black or American Indian & Alaska Native, the COVID-19 infection rate in log scale increased significantly. With the higher of segregation index, the lower of COVID-19 infection rate in log scale (b = -0.00457, 95% CI: -0.00723, - 0.00192). The percentage of Hispanic in the population showed a negative correlation with the infection rate of COVID-19 in log scale (b = -0.01236, 95% CI: -0.01957, -0.00515).
Reports and other publications	41	Chen & Krieger [60]	Revealing the unequal burden of COVID-19 by income, race/ethnicity, and household crowding: US county vs. ZIP code analyses	USA	21.04.2020	Ecological study	Population of the USA, Illinois, New York	Data until 16.04.2020	Area-based socioeconomic measures (ABSM)	Incidence, mortality on county level	Highest death rates were observed in counties with the highest poverty; highest incidende in zip codes with highest ABSM.
	42	Chen et al. [61]	COVID-19 and the unequal surge in mortality rates in Massachusetts, by city/town and ZIP Code measures of poverty, household crowding, race/ethnicity, and racialized economic segregation	USA	09.05.2020	Ecological study	Population of Massachusetts	01.01. - 15.04.2020	Area-based socioeconomic measures (ABSM)	Excess death rate linked to COVID-19	Surge in excess death rates, both relative and absolute, was evident starting in early April, and was greater in city/towns and ZCTAs with higher poverty, higher household crowding, higher percentage of populations of color, and higher racialized economic segregation.
	43	Public Health England [78]	Disparities in the risk and outcomes of COVID-19	United Kingdom	02.06.2020	Ecological study	Population of England and Wales		Index of Multiple Deprivation (IMD), occupation		The trend in the number of diagnosed cases by deprivation quintile shows that cases in the least deprived group peaked earlier and lower than other groups and at 13 May, the cumulative number of cases and diagnosis rate was highest in the most deprived death. The trend in the number of diagnosed cases by deprivation quintile shows that cases in the least deprived group peaked earlier and lower than other groups and at 13 May, the cumulative number of cases and diagnosis rate was highest in the most deprived quintile. For three occupations the relative increase in deaths in 2020 was significantly higher than the average of 1.5: Caring Personal Services, Elementary Security Occupations, and Road Transport Drivers. Of these groups, the biggest increase was for Elementary Security Occupations, where deaths were 2.3 times higher in 2020 than in the same period in 2014 to 2018. Within these groups, there were three occupational ‘unit groups’ where the increase in deaths in 2020 was significantly higher than the increase for everyone aged 20 to 64. These were nursing auxiliaries and assistants, security guards and related occupations, and taxi and cab drivers and chauffeurs. The largest absolute increase was for workers in Caring Personal Services. There were 760 deaths from all causes among these workers in the period 21 March to 8 May 2020 for people aged 20 to 64. This is 346 more than in the same period in 2014 to 2018 and 74% had COVID-19 recorded as a cause of death. For workers in Construction and Building Trades, the number of deaths related to COVID-19 was slightly higher than the number of excess deaths. This indicates that deaths from other causes have gone down which may be due to a reduced risk of occupational related injuries over this time period.
	44	Office for National Statistics [79]	Deaths involving COVID-19 by local area and socioeconomic deprivation: deaths occurring between 1 March and 31 May 2020	England and Wales	12.06.2020	Ecological study	46,687 deaths linked to COVID-19	01.03. - 31.05.2020	Index of Multiple Deprivation (IMD)	Mortality	Looking at deaths involving the coronavirus (COVID-19), in England, the rate for the least deprived area (Decile 10) was 58.8 deaths per 100,000 population and the rate in the most deprived area (Decile 1) was 128.3 deaths per 100,000 population; this is 118% higher than the least deprived area. In the least deprived area, the age-standardised mortality rate for all deaths was 242.6 deaths per 100,000 population. In the most deprived area, the age-standardised mortality rate for all deaths was 92.2% higher than that of the least deprived, at 466.2 deaths per 100,000 population. The most deprived fifth of areas (quintile) in Wales had a rate of 109.5 deaths involving the coronavirus (COVID-19) per 100,000 population; this was nearly twice as high as the least deprived areas (57.5 deaths per 100,000 population) and over twice as high as the lowest mortality rate in Quintile 4 (50.5 deaths per 100,000 population).
	45	Office for National Statistics [84]	Coronavirus (COVID-19) related deaths by occupation, England and Wales: deaths registered up to and including 20 April 2020	England and Wales	11.05.2020	Analysis of official statistics	2,494 deaths in the working-age population of England and Wales	Until 20.04.2020	Occupation	Mortality	Compared with the rate among people of the same sex and age in England and Wales, men working in the lowest skilled occupations had the highest rate of death involving COVID-19, with 21.4 deaths per 100,000 males (225 deaths); men working as security guards had one of the highest rates, with 45.7 deaths per 100,000 (63 deaths). Men and women working in social care, a group including care workers and home carers, both had significantly raised rates of death involving COVID-19, with rates of 23.4 deaths per 100,000 males (45 deaths) and 9.6 deaths per 100,000 females (86 deaths). Among men, a number of other specific occupations were found to have raised rates of death involving COVID-19, including: taxi drivers and chauffeurs (36.4 deaths per 100,000); bus and coach drivers (26.4 deaths per 100,000); chefs (35.9 deaths per 100,000); and sales and retail assistants (19.8 deaths per 100,000).
	46	Intensive Care National Audit & Research Centre [80]	ICNARC report on COVID-19 in critical care 12 June 2020	England, Wales and Northern Ireland	12.06.2020	Analysis of registry data / Ecological study	9,777 patients from 289 ICU in England, Wales and Northern Ireland	Until 11.06.2020	Index of Multiple Deprivation (IMD)	Admission to Intensive Care due to COVID-19	24,4 % of all COVID-19 ICU patients came from localities (postcode) in 4th Quintile and 25,3% from 5th Quintile of the distribution of the IMD.

* References can be found in the article Wachtler B, Michalski N, Nowossadeck E, Diercke M, Wahrendorf M et al. (2020) Socioeconomic inequalities and COVID-19 – A review of the current international literature. Journal of Health Monitoring 5(S7): 3–17 of the Journal of Health Monitoring.

ABSM = area-based socioeconomic measures, aOR = adjusted odds ratio, aRR = absolute risk reduction, BAME = black and minority ethnic, CI = confidence interval, GDP = gross domestic product, HR = hazard ratio, ICU = intensive care unit, IMD = Index of Multiple Deprivation, IRR = incident rate ratio, MRR = median rate ratio, NHS = National Health Service, OR = odds ratio, RR = relative risk, SD = standard deviation, SVI = Social Vulnerability Index, ZCTA = ZIP Code Tabulation Area

